# Proteome Expression Signatures: Differences between Orbital and Subcutaneous Abdominal Adipose Tissues

**DOI:** 10.3390/life14101308

**Published:** 2024-10-15

**Authors:** Noam Castel, Edward Vitkin, Sharon Shabo, Ariel Berl, Julia Wise, Amir Duenyas, Eliyahu Michael Aharon Cohen, Alexander Golberg, Avshalom Shalom

**Affiliations:** 1 Department of Plastic Surgery, Meir Medical Center, Kfar Sava 4428164, Israelarielberl23@gmail.com (A.B.); 2 Department of Environmental Studies, Porter School of Environment and Earth Sciences, Tel Aviv University, Tel Aviv 6997801, Israelcohen11@mail.tau.ac.il (E.M.A.C.);; 3 School of Medicine, Faculty of Medical and Health Sciences, Tel Aviv University, Tel Aviv 6997801, Israel

**Keywords:** orbital fat, abdominal subcutaneous fat, proteomics, differential expression

## Abstract

Differences between orbital and subcutaneous abdominal fat in the same patient have been noted but not formally investigated, previously. The objective of this research was to compare the differential expression of protein profiles in subcutaneous abdominal and orbital adipose tissues. In this cross-sectional, observational study, orbital fat tissue was sampled from 10 patients who underwent blepharoplasty and agreed to provide a small sample of subcutaneous abdominal fat. Shotgun mass spectrometry was performed on the extracted proteome. Data were analyzed using protein appearance patterns, differential expression and statistical enrichment. Protein analysis revealed significant differences in proteomics and differential expression between the orbital and subcutaneous abdominal adipose tissues, which presented five proteins that were uniquely expressed in the orbital fat and 18 in the subcutaneous abdominal fat. Gene Ontology analysis identified significantly different cellular processes and components related to the extracellular matrix or basement membrane components. This analysis shows the differences between orbital and subcutaneous abdominal fat found in proteomics differential expression, uniquely expressed proteins, and cellular processes. Further research is needed to correlate specific proteins and cellular processes to the mechanism of fat accumulation and obesity.

## 1. Introduction

Obesity is a major concern in the western world. According to the World Health Organization, since 1975, the incidence of obesity has increased nearly three-fold. In 2022, over 2.5 billion people at the age of 18 years or older (39% of the world population) were overweight, and over 890 million adults (13%) had obesity [1].

Interestingly, histological observation of adipose tissue reveals that not all adipocytes are the same size [2]. Among people who meet the definitions of overweight and obese, the orbital area does not seem to accumulate fat in the same manner as other regions of the body, even though the existence of orbital fat pads is well described. The distinctiveness of orbital fat and nasal orbital fat is a common clinical observation by plastic surgeons during blepharoplasty. Moreover, its light color and characteristics of carotenoid content were previously described, as well as its fibrous tissue and triglyceride deposits, which differentiate it from other adipose tissues [3].

Several studies have established some differences between orbital and subcutaneous abdominal fat. Nepali et al. [4] and Afanas’eva et al. [5] found unique and overexpressed surface antigens and cytokines in mesenchymal stromal cells extracted from orbital and subcutaneous abdominal fat. Wang et al. [6] further noted that subcutaneous eyelid fat originated from the ectoderm embryonal layer and not the endodermal layer, and demonstrated its regenerative properties compared with abdominal subcutaneous fat. Sun et al. [2] compared orbital and subcutaneous abdominal fat from the same patient, histologically. They found that the orbital fat contained smaller adipocytes surrounded by dense fibrous tissues compared to the adipocytes in the abdominal subcutaneous fat. This observation of smaller adipocytes was attributed to low metabolic activity [7]. These studies demonstrating the uniqueness of orbital fat, combined with clinical observations of orbital fat as a “non-fat-gaining tissue”, led us to explore and compare the differences in protein expression and metabolic pathways between orbital and subcutaneous abdominal fat. The rationale for comparing these two types of fat was based on the literature mentioned above, and was possible because as plastic surgeons performing blepharoplasties, we had access to orbital fat tissue. Subcutaneous abdominal tissue is easily accessible, requiring only a small, superficial tissue sample that was easily obtained.

A better understanding of the different ways in which the two tissues accumulate fat may shed light on fat gaining mechanisms. Comparing molecular components between the orbital and subcutaneous abdominal fat tissues may reveal some of the factors involved in the accumulation of subcutaneous abdominal fat compared to orbital fat. These components could serve as molecular targets for future medications or other molecular-oriented treatments for obesity.

The goal of this cross-sectional, observational study was to evaluate and compare protein expression and cellular processes in both orbital and subcutaneous abdominal fat, to improve current knowledge of the metabolic pathways that account for the differences between these morphologically different tissues.

## 2. Materials and Methods

### 2.1. Participants

Ten patients who underwent upper blepharoplasty at Meir Medical Center, Israel during the period March 2021–March 2022 provided tissue samples and a small sample of subcutaneous abdominal fat. Demographic data collected included age, sex, medical history, medical treatment, and body mass index (BMI, kg/m^2^) (Table 1). Those with an active malignancy, on immunosuppressive treatment or chemotherapy, and patients who were pregnant at the time of the procedure were excluded.

### 2.2. Surgical Procedure

Patients underwent upper blepharoplasty under general anesthesia. As part of the procedure, the surgeons removed excess skin from the upper eyelid, as well as excess orbital fat from the medial and central compartments. The fat samples were stored in a sterile vessel.

During the same procedure, a sample of subcutaneous abdominal fat was taken using a 16G true-cut biopsy needle (Merit Medical, South Jordan, UT, USA). The orbital and abdominal subcutaneous fat samples were processed for protein isolation 20–30 min after excision.

### 2.3. Protein Identification and Quantification

#### 2.3.1. Protein Isolation

Tissues were homogenized using the bead beater (Biospec, Bartlesville, OK, USA) in 2 mL tubes and filled to one-third with 2 mm zirconia (Sarstedt, Germany). Using the EZ-RNA II kit (Biological Industries Ltd., Beit Ha’emek, Israel), proteins were isolated from the extract, without the use of homogenizing solutions. Phase separation solutions of 0.2 mL of water-saturated phenol and 0.045 mL of bathocuproine buffer were added directly into the tubes. After the bead beating step, proteins were precipitated with isopropanol, followed by washing with guanidine hydrochloride in 95% ethanol. After air drying, the protein pellets underwent proteomic analysis, described in the next section.

#### 2.3.2. Proteolysis

The protein pellets were resuspended in a solution of 10 mM dithiothreitol, 8.5 M urea, and 400 mM ammonium bicarbonate, and were then vortexed, sonicated for 5 min at 90% with 10-10 cycles, and centrifuged. Bradford readings were used to estimate the amount of protein extracted. Next, 20 µg of protein from each sample was reduced at 60 °C for 30 min. This was then modified with 37.5 mM iodoacetamide in 100 mM ammonium bicarbonate for 30 min at room temperature, in the dark. This step was followed by digestion in 66.6 mM ammonium bicarbonate and 1.5 M urea, with modified trypsin (Promega, Madison, WI, USA) at an enzyme-to-substrate ratio of 1:50, overnight at 37 °C. It was digested a second time with trypsin for 4 h at 37 °C.

#### 2.3.3. Mass Spectrometry Analysis

Using a homemade C18 stage tip, the tryptic peptides were desalted, dried and resuspended in 0.1% formic acid. The peptides were loaded in solvent A (0.1% formic acid in water) on a homemade capillary column (30 cm, 75-micron ID) packed with Reprosil C18-Aqua (Dr. Maisch, GmbH, Ammerbuch-Entringen, Germany). The peptide mixture was resolved with a 5% to 28% linear gradient of solvent B (80% acetonitrile with 0.1% formic acid) for 180 min, followed by a gradient of 15 min of 28% to 95% and 25 min at 95% B with 0.1% formic acid in water, at a flow rate of 0.15 μL/min. A Q-Exactive HFX mass spectrometer (Thermo Fisher Scientific, Waltham, MA, USA), set at the positive mode was used to perform a complete mass spectrometry (MS) scan. This was followed by a dissociation of the 30 most dominant ions selected from the first MS scan, induced by higher energy collision. The data were analyzed using MaxQuant software 1.5.2.8 (M. Mann’s group) compared to the human proteome obtained from the Uniprot database, with a 1% false discovery rate (FDR). The data were quantified with the same software using label-free analysis, based on extracted ion currents from the peptides. This enabled quantitation from every liquid chromatography/MS scans for each of the peptides identified in the experiments.

### 2.4. Bioinformatic Data Analysis

#### 2.4.1. Analysis of Protein Appearance Patterns

Based on the raw MS data obtained from the MaxQuant program, proteins appearing in the samples were assigned a value of 1′ if they were observed with any positive intensity and assigned 0′, if otherwise. If proteins were observed only among samples with this condition, they were defined as unique for the specific condition. Unique proteins that appeared in at least 40% of patients (4 patients) were addressed as of interest and reported in Table 2 and Table 3. The differential analysis was calculated as the ratio of the observed to expected proteins for a given *p*-value threshold. The highly ranked, uniquely expressed proteins were manually investigated for relevant interactions and pathways in the Kyoto Encyclopedia of Genes and Genomes database (KEGG DB), PHAROS DB, IntAct DB, and other public sources [8,9,10].

#### 2.4.2. Differential Expression Analysis

The differential expression of proteins between orbital and subcutaneous abdominal adipose tissues was analyzed using the following: (i) a parametric student t-test (*scipy.stats.ttest_rel*), and (ii) nonparametric Wilcoxon signed rank (*scipy.stats.wilcoxon*) tests for related paired samples, and with (iii) a proportion-based analysis of appearance patterns, that compared the protein appearance frequencies between two groups [11]. The latter test was also used to calculate the statistical significance of the uniqueness of the observed proteins (Table 2 and Table 3). The Benjamini–Hochberg procedure (*scipy.stats.false_discovery_control*) was used to correct for multiple testing of the resulting *p*-values. All results are reported in Tables S1 and S2.

Differentially expressed proteins were sorted according to the minimum rank resulting from the differential expression tests and fed into Gene Ontology (GO) analysis with the GOrilla tool [12] (Table S3 contains the exact inputs) to identify statistically significant overrepresentations of specific cellular processes, functions, and components among the most differentially changed proteins. Full outputs are available in Table S4.

## 3. Results

Study participants included seven women and three men, ages 68 to 84 years (mean age was 72.3), who underwent upper eyelid blepharoplasty and provided a small sample of subcutaneous abdominal fat, taken using a true-cut biopsy needle. The proteomic profile of each sample was measured, resulting in 2773 proteins that were observed at least once. In total, this study is based on proteomes measured in over 10 pairs of fat samples. Table 1 describes the demographics of the 10 participants.

### 3.1. Differences between Proteomes Measured in Orbital and Subcutaneous Abdominal Fat Tissues

**Figure 1:**
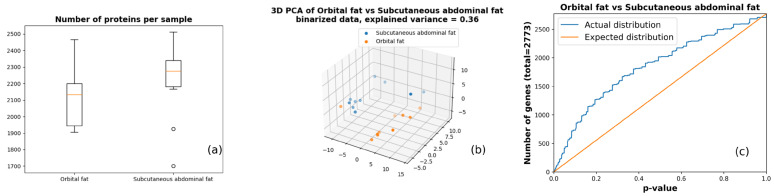
Sample-level difference between the orbital and subcutaneous abdominal fat tissues. (**a**) Distribution of the number of proteins identified per tissue sample. (**b**) Three-dimensional PCA (Principal Component Analysis) plot of protein binary appearance patterns. (**c**) Overabundance plot for differentially expressed proteins (based on Wilcoxon signed rank test).

Proteomes gathered from the 10 pairs of samples were compared according to the protein binary appearance patterns to focus on the most obvious changes in fat protein dynamics. The comparison showed a clear difference in the number of proteins observed between orbital and subcutaneous abdominal fat in each sample (Figure 1a), in the spatial distribution of 3D Principal Component Analysis (PCA) projection of the protein binary appearance patterns (Figure 1b), and in the overabundance analysis comparing the number of observed and expected differentially expressed proteins (Figure 1c). The latter was based on a paired, nonparametric Wilcoxon signed rank test and resulted in 83 proteins with *p* < 0.01 (FDR = 0.33) and 413 proteins with *p* = 0.05 (FDR = 0.34).

### 3.2. Differentially Expressed Proteins in Orbital and Subcutaneous Abdominal Fat Tissues

**Figure 2:**
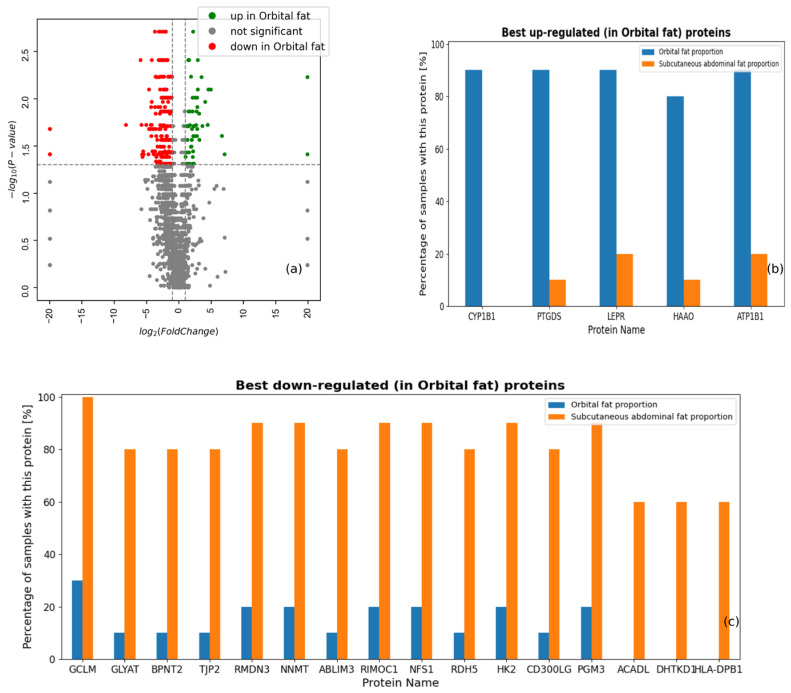
Protein-level differences between the proteomes in orbital and subcutaneous abdominal fat tissues. (**a**) Volcano plot of proteins based on Wilcoxon signed rank test. (**b**) Proteins with the most significantly up-regulated binary appearance patterns in orbital fat. (**c**) Proteins with the most significantly down-regulated binary appearance patterns in abdominal fat.

A total of 2773 proteins were detected with positive intensity in at least one sample, using shotgun mass spectrometry (Table S1). Differential expression analysis of specific proteins identified many potentially interesting biomarker candidates (Figure 2a). In agreement with Figure 1a, we observed more proteins that were significantly under-expressed in orbital fat tissue. For example, in Figure 2b,c, genes DHTKD1 (probable 2-oxoglutarate dehydrogenase E1 component DHTKD1), ACADL (long-chain specific acyl-CoA dehydrogenase), and HLA-DPB1 (HLA class II histocompatibility antigen, DP beta 1 chain) were observed in 60% of subcutaneous abdominal fat samples and not in the orbital fat samples, while the *Cytochrome P450 1B1* gene was observed in 90% of orbital fat samples and was not observed in abdominal fat samples.

### 3.3. Proteins Uniquely Detected in Subcutaneous Abdominal and Orbital Fat Tissues

Further exploration of the differences between the orbital and subcutaneous abdominal fat revealed five proteins in the orbital fat (in at least 40% of patients) that were not in the subcutaneous abdominal fat (Table 2), and 18 proteins in the subcutaneous abdominal fat that were not in the orbital fat (Table 3). For example, Cytochrome P450 1B1 was detected in the orbital fat of nine out of ten participants and in none of the abdominal fat samples. Based on the KEGG pathway analysis, Cytochrome P450 1B1 participates in seven metabolic pathways, including steroid hormone biosynthesis and tryptophan metabolism (Table 2). In addition, mitochondrial long-chain specific acyl-CoA dehydrogenase was found in the subcutaneous abdominal fat of six out of ten participants and in none of the orbital fat samples. Based on the KEGG, long-chain specific acyl-CoA dehydrogenase participates in four metabolic pathways: fatty acid degradation, metabolic pathways, fatty acid metabolism, and the peroxisome proliferator-activated receptor (PPAR) signaling pathway (Table 3).

### 3.4. Gene Ontology Analysis of the Proteomes Extracted from Orbital and Subcutaneous Abdominal Adipose Tissues

Gene ontology was analyzed to further identify cellular processes, cellular functions and cellular components. All 2773 proteins identified were sorted according to the minimum rank resulting from differential expression tests (proportion, student t-test and Wilcoxon signed rank-sum, Table S2). The cellular processes, functions, and components were fed into GO analysis using the GOrilla tool [12,13,14]. We identified two significantly changed GO cellular processes, one GO cellular function and three GO cellular components that were significantly enriched (FDR q-value < 1 × 10^−3^, which is a *p*-value after the correction for multiple testing, as reported by the GOrilla tool) (Table 4). All are related to the extracellular matrix organization and membranal components of the fat tissue.

## 4. Discussion

This study compared the protein expression of orbital versus subcutaneous abdominal adipose tissues. The analysis found significant differences in the proteomes, differential expression and cellular processes between the two types of tissue.

We found a clear difference in the proteomes between the orbital and subcutaneous abdominal fat tissues (Figure 1a) and illustrated the non-random distribution of the proteins present in the tissues examined (Figure 1c), confirming that the proteomes of the orbital and subcutaneous abdominal fat tissues are indeed significantly different.

The differential expression between the fat tissues (Figure 2) shows more proteins that are significantly under-expressed in orbital fat tissue, while some proteins were uniquely expressed in only one type of the fat tissues examined. Five proteins were exclusively expressed in orbital but not abdominal fat. Among them, Cytochrome P450 1B1 stood out because it was expressed only in the orbital fat tissue of nine of the ten patients. Cytochrome P450 1B1 is a widely studied protein, mentioned in the literature in several contexts. Its main functions are attributed as the hydroxylation of estrogen, and retinol and melatonin metabolism. It is also noted for its crucial role in glaucoma, where different mutations determine the severity of the glaucoma phenotype [15,16,17]. Cytochrome P450 1B1 has a very important role in the prevention of glaucoma and its expression in the orbital fat might be due to its presence in the retinal vascular cells and astrocytes [18].

Several studies correlated Cytochrome P450 1B1 with obesity, where accumulating data indicate that genetic manipulations of Cytochrome P450 1B1 can decrease adipogenesis and prevent obesity. Li et al. [16] showed that the disruption of Cytochrome P450 1B1 in mice suppresses obesity induced by a high-fat diet. In addition, expression of the obesity markers hepatic stearoyl-CoA desaturase 1 and LPC 18:0 were significantly decreased in Cytochrome P450 1B1 null mice. Similarly, Liu et al. [19] indicated that Cytochrome P450 1B1 deficiency can attenuate obesity in mice that was induced by a high-fat diet and improve glucose tolerance. In a review of 49 genome-wide sequencing experiments related to obesity that included 16186 genes, English and Butte [20] showed that Cytochrome P450 1B1 was the third highest scoring gene associated with obesity. Considering initial observations that orbital adipose tissue does not accumulate fat as does subcutaneous abdominal tissue, the unique expression of an enzyme linked to obesity in the orbital fat tissue was somewhat surprising. Moreover, when we examined the relation between Cytochrome P450 1B1 expression and BMI in our study population, we found a positive correlation between the protein expression and the BMI of the patients (R^2^ = 0.35). Although our cohort included only 10 patients, these preliminary findings are intriguing and require further investigation with a larger population. However, it should be considered that although we analyzed actual fat tissues, the extracellular matrix was also processed.

Eighteen proteins were uniquely expressed in subcutaneous abdominal fat. Long-chain specific acyl-CoA dehydrogenase, one of the acyl-CoA dehydrogenases that catalyze the first step of mitochondrial fatty acid beta-oxidation, was uniquely expressed in the subcutaneous abdominal fat of six out of ten patients. This is an aerobic process allowing the production of energy from fats [21].

GO analysis identified two cellular processes, one cellular function and three cellular components that were significantly enriched, whereas all cellular processes were found to be connected to extracellular matrix or basement membrane components. Previous studies examining orbital and abdominal fat have addressed intracellular components [2], adipocyte morphology [4], or adipose-derived mesenchymal antigens [3]. To our knowledge, this is the first study to relate extracellular processes and basement membrane components to the differences between orbital and subcutaneous abdominal fat. This observation is important because it highlights how different fat deposits (orbital vs. subcutaneous abdominal fat) may be regulated by their surrounding environments. Understanding how extracellular processes and basement membrane components contribute to these differences could shed light on why certain types of fat behave differently in metabolic diseases.

While this study successfully identifies significant differences between the proteomes of orbital and subcutaneous abdominal adipose tissues, it has several limitations that must be acknowledged.

The cohort included only 10 patients, with varied demographic characteristics (age, sex, BMI, and medical background). A larger study group could potentially reinforce the results and enable these variations to be addressed in the data analysis. Additionally, the research was observational; hence, it could not indicate one mechanism or molecular component as a cause for the morphological differences observed between orbital and subcutaneous abdominal adipocytes but instead provided a broader picture which needs further attention and research. Additional studies are needed to find linkages between the observations reported here and the molecular and biological mechanisms responsible for the differences between the adipose tissues.

Additional limitations involve the analytical methods used. First, although mass spectrometry (MS) is a powerful tool for large-scale protein identification and quantification, it has inherent limitations, especially when it comes to distinguishing between homologous proteins, isoforms, and proteoforms [21,22,23,24]. Homologous proteins share high sequence similarity, and their differentiation based solely on peptide mass or fragmentation patterns can be challenging [25,26]. Similarly, protein isoforms, which arise from alternative splicing, and proteoforms, which result from post-translational modifications (PTMs), often exhibit minor differences that may not be adequately resolved via standard MS workflows [21,22,23].

This study relied on a label-free quantification approach, which, while highly effective for detecting proteins in complex biological samples, does not easily differentiate between these closely related protein species [26,27,28,29]. As a result, some proteins detected in both tissue types may represent different isoforms or proteoforms, which are difficult to distinguish based on peptide fragmentation data alone. This is particularly relevant for proteins involved in complex regulatory pathways, where subtle changes in PTMs or isoforms can have significant biological implications that might be overlooked by our current methodology.

Moreover, MS-based proteomics often face difficulties when analyzing low-abundance proteins [27,28,29]. Certain functionally relevant proteins may be present at concentrations below detectable limits, leading to an incomplete picture of the proteome. The data we present here likely reflects this limitation, and future research incorporating enrichment strategies for low-abundance proteins or employing complementary techniques, such as targeted proteomics, may provide deeper insights into the differential protein expression between these adipose tissues [25,26,28,29].

Finally, as the study is cross-sectional in nature, it provides a snapshot of the proteome at a single time point. Dynamic changes in protein expression and modifications in response to environmental or physiological factors may not be fully captured here. Future longitudinal studies, integrating other -omics approaches like lipidomics or metabolomics, may help provide a more comprehensive understanding of the metabolic differences between these adipose tissues.

## 5. Conclusions

This study identified differences between orbital and subcutaneous abdominal fat on the levels of proteomics, differential expression, uniquely expressed proteins, and cellular processes. These preliminary results add to our knowledge of adipose tissues in the human body and about the cellular components that might be responsible for variations in fat accumulation in different adipose tissues. Further research is needed to correlate specific proteins and cellular processes to the mechanism of fat accumulation, and hopefully contribute to the prevention and treatment of obesity.

## Figures and Tables

**Table 1:** Demographics of the 10 research participants.

Patient	Age	BMI	Sex	Medical Background
1	68	23.8	F	Hypertension, hypercholesteremia, glaucoma
2	71	27.3	F	Hypertension
3	69	19.7	F	Hypothyroidism, osteoporosis
4	75	29.2	F	Type II diabetes, hypertension, hypothyroidism, dyslipidemia
5	73	30.5	F	Asthma
6	68	23.8	F	Dyslipidemia
7	71	27.3	F	Spinal muscular atrophy
8	69	19.7	M	Gout
9	75	29.2	M	Healthy
10	84	28.4	M	Hypertension, type II diabetes, hypercholesterolemia, ischemic heart disease

BMI, body mass index (kg/m^2^).

**Table 2:** Uniquely detected proteins in orbital fat (in at least 40% of patients).

Protein ID	Protein Name	% of Patients	*p*-Value	Database	Pathways
Q16678	Cytochrome P450 1B1	90%	5.23 × 10^−5^	KEGG	Steroid hormone biosynthesis, tryptophan metabolism, metabolism of xenobiotics by Cytochrome P450, ovarian steroidogenesis, chemical carcinogenesis—DNA adducts, MicroRNAs in cancer, chemical carcinogenesis—receptor activation, chemical carcinogenesis—reactive oxygen species.
O94788	Retinal dehydrogenase 2	50%	9.82 × 10^−3^	KEGG	Metabolic pathways, retinol metabolism
P02812	Basic salivary proline-rich protein 2	40%	2.53 × 10^−2^	KEGG	Salivary secretion
Q13740	CD166 antigen	40%	2.53 × 10^−2^	KEGG	Cell adhesion molecules
P08582	Melanotransferrin	40%	2.53 × 10^−2^	PHAROS	Protein metabolism, post-translational protein modification, post-translational modification: synthesis of GPI-anchored proteins, post-translational protein phosphorylation, regulation of insulin-like growth factor transport and uptake by insulin-like growth factor binding proteins

**Table 3:** Uniquely detected proteins in subcutaneous abdominal fat (in at least 40% of patients).

Protein ID	Protein Name	% of Patients	*p*-Value	Database	KEGG Pathway
P28330	Long-chain specific acyl-CoA dehydrogenase, mitochondrial	60%	3.41 × 10^−3^	KEGG	Fatty acid degradation, fatty acid metabolism, metabolic pathways, PPAR signaling pathway
Q96HY7	Probable 2-oxoglutarate dehydrogenase E1 component DHKTD1, mitochondrial	60%	3.41 × 10^−3^	KEGG	Lysine degradation, tryptophan metabolism, lipoic acid metabolism, metabolic pathways, biosynthesis of secondary metabolites, 2-oxocarboxylic acid metabolism
P04440	HLA class II histocompatibility antigen, DP beta 1 chain	60%	3.41 × 10^−3^	UNIPROT	Downstream TCR signaling, CD3 and TCR zeta chain phosphorylation, translocation of ZAP-70 to immunological synapse, generation of second messenger molecules, Presentation of MHC class II antigens
P47712	Cytosolic phospholipase A2	50%	9.82 × 10^−3^	see 23 pathways in the link	https://www.genome.jp/dbget-bin/www_bget?hsa:5321 (accessed on 10 May 2024)
Q8IWW8	Hydroxyacid-oxoacid transhydrogenase, mitochondrial	50%	9.82 × 10^−3^	HMDB	Oncogenic action of D-2-hydroxyglutarate in hydroxygluaricaciduria, oncogenic action of L-2-hydroxyglutarate in hydroxygluaricaciduria
Q10713	Mitochondrial-processing peptidase subunit alpha	50%	9.82 × 10^−3^	PHAROS	Mitochondrial calcium ion transport, 3-phosphoinositide degradation, mitochondrial protein import, processing of SMDT1, protein localization, small molecule transport
Q6GTX8; Q6ISS4	Leukocyte-associated immunoglobulin-like receptor 1	50%	9.82 × 10^−3^	PHAROS	Adaptive immune system (R-HSA-1280218), immune system (R-HSA-168256), immunoregulatory interactions between lymphoid and non-lymphoid cells (R-HSA-198933), innate immune system (R-HSA-168249), neutrophil degranulation (R-HSA-6798695)
Q13424	Alpha-1-syntrophin	50%	9.82 × 10^−3^	KEGG	TGF-beta signaling pathway, ECM-receptor interaction, Renin-angiotensin system, JAK-STAT signaling pathway
Q5TFE4	5-nucleotidase domain-containing protein 1	50%	9.82 × 10^−3^		Not found
Q92552	28S ribosomal protein S27, mitochondrial	50%	9.82 × 10^−3^		Mitochondrial translation
Q96GG9	DCN1-like protein 1	50%	9.82 × 10^−3^		Neddylation, post-translational protein modification
Q14008	Cytoskeleton-associated protein 5	40%	2.53 × 10^−2^	See super pathways and contained pathways in the link	https://www.genecards.org/cgi-bin/carddisp.pl?gene=CKAP5#pathways_interactions (accessed on 10 May 2024)
Q6L8Q7	2,5-phosphodiesterase 12	40%	2.53 × 10^−2^	PHAROS	Antiviral mechanism by IFN-stimulated genes, cytokine signaling in immune system, immune system, interferon signaling, OAS antiviral response
P10619	Lysosomal protective protein	40%	2.53 × 10^−2^	KEGG	Other glycan degradation, glycosaminoglycan degradation, SNARE interactions in vesicular transport, autophagy—animal, endocytosis
P27918	Properdin	40%	2.53 × 10^−2^	PHAROS	Complement cascade, immunoregulatory interactions between a lymphoid and a non-lymphoid cell, cell recruitment (pro-inflammatory response)
Q9BYT8	Neurolysin, mitochondrial	40%	2.53 × 10^−2^		Renin-angiotensin system
O94925	Glutaminase kidney isoform, mitochondrial	40%	2.53 × 10^−2^	KEGG	Citrate cycle (TCA cycle), pyrimidine metabolism, alanine, aspartate and glutamate metabolism, arginine and proline metabolism, D-amino acid metabolism, nitrogen metabolism
P06454	Prothymosin alpha	40%	2.53 × 10^−2^	PHAROS	Validated targets of C-MYC transcriptional activation

**Table 4:** GO terms identified as significantly different between orbital and subcutaneous abdominal fat tissues. FDR (False Discovery Rate) q-value corresponds to *p*-value after the correction for multiple testing, as reported by the GOrilla tool [12,13,14].

GO Class	GO Term	Description	*p*-Value	FDR q-Value
PROCESS	GO:0043062	Extracellular structure organization	6.39 × 10^−8^	6.16 × 10^−4^
PROCESS	GO:0030198	Extracellular matrix organization	1.28 × 10^−7^	6.17 × 10^−4^
FUNCTION	GO:0005201	Extracellular matrix structural constituent	1.27 × 10^−7^	3.15 × 10^−4^
COMPONENT	GO:0005604	Basement membrane	2.83 × 10^−7^	3.76 × 10^−4^
COMPONENT	GO:0031224	Intrinsic component of membrane	2.96 × 10^−7^	1.97 × 10^−4^
COMPONENT	GO:0016021	Integral component of membrane	5.80 × 10^−7^	2.57 × 10^−4^

## Data Availability

All data are included in the text and Supplementary Materials.

## Supplementary Materials

The following supporting information can be downloaded at https://www.mdpi.com/article/10.3390/life14101308/s1; Table S1: Raw measurements.xlsx; Table S2: Differential expression results.xlsx; Table S3: GOrilla inputs.csv; Table S4: GOrilla outputs.xlsx; Figure S1: Correlations between Cytochrome P450 1B1 expression and BMI.

## Abbreviations

BMI body mass index (kg/m^2^), DB database, FDR false discovery rate, GO Gene Ontology, KEGG Kyoto Encyclopedia of Genes and Genomes, MS mass spectrometry, PCA Principal Component Analysis, PPAR peroxisome proliferator-activated receptor.
