# Supplementary material for: Proteome Expression Signatures: Differences between Orbital and Subcutaneous Abdominal Adipose Tissues
Source: Life (Basel). 2024 Oct 15;14(10):1308. doi: 10.3390/life14101308 (PMC11509502; doi:10.3390/life14101308)
Supplement: Supplementary file 1 [file life-14-01308-s001.zip › life-3154073-supplementary/Figure S1. Correlation between Cyp450 1b1 expression and BMI.pdf]

Figure S1: Correlation between Cytochrome P540 1B1 expression with the BMI of each patient.

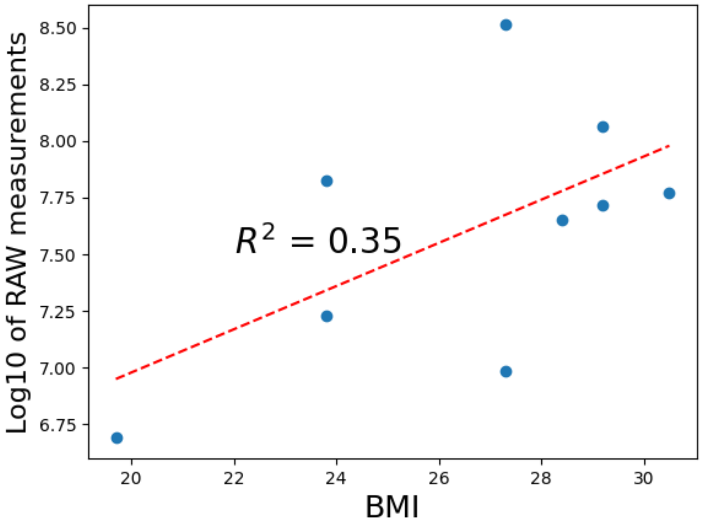

Cytochrome P540 1B1 expression shows posiitve correlation with the BMI of each patient.
